# High-throughput sequence analysis of variants of human cytomegalovirus strains Towne and AD169

**DOI:** 10.1099/vir.0.013250-0

**Published:** 2009-10

**Authors:** Amanda J. Bradley, Nell S. Lurain, Peter Ghazal, Urmi Trivedi, Charles Cunningham, Katarina Baluchova, Derek Gatherer, Gavin W. G. Wilkinson, Derrick J. Dargan, Andrew J. Davison

**Affiliations:** 1 MRC Virology Unit, Institute of Virology, Church Street, Glasgow G11 5JR, UK; 2 Department of Immunology and Microbiology, Rush University Medical Center, 1653 West Congress Parkway, Chicago, IL 60612, USA; 3 Division of Pathway Medicine, University of Edinburgh Medical School, Chancellor's Building, 49 Little France Crescent, Edinburgh EH16 4SB, UK; 4 The Gene Pool, Ashworth Laboratories, Institute of Evolutionary Biology, King's Buildings, Edinburgh EH9 3JT, UK; 5 Department of Medical Microbiology, Tenovus Building, School of Medicine, Cardiff University, Heath Park, Cardiff CF14 4XX, UK

## Abstract

The genomes of commonly used variants of human cytomegalovirus (HCMV) strains Towne and AD169 each contain a substantial mutation in which a region (U_L_/*b′*) at the right end of the long unique region has been replaced by an inverted duplication of a region from the left end of the genome. Using high-throughput technology, we have sequenced HCMV strain Towne (ATCC VR-977) and confirmed the presence of two variants, one exhibiting the replacement in U_L_/*b′* and the other intact in this region. Both variants are mutated in genes RL13, UL1, UL40, UL130, US1 and US9. We have also sequenced a novel AD169 variant (varUC) that is intact in U_L_/*b′* except for a small deletion that affects genes UL144, UL142, UL141 and UL140. Like other AD169 variants, varUC is mutated in genes RL5A, RL13, UL36 and UL131A. A subpopulation of varUC contains an additional deletion affecting genes IRS1, US1 and US2.

Human cytomegalovirus (HCMV; species *Human herpesvirus 5*) was first isolated over 50 years ago (Craig *et al.*, 1957; Rowe *et al.*, 1956; Smith, 1956). The most widely used laboratory strains are Towne (Plotkin *et al.*, 1975) and AD169 (Rowe *et al.*, 1956). Both have been distributed widely and developed as vaccine candidates, and over the years their detailed histories have become obscure. Moreover, the fact that the biological properties of these strains are not conserved between stocks (Brown *et al.*, 1995) demonstrates the existence of genetic variants, and this may affect the interpretation of experimental studies. This paper contributes to the characterization of variants present in the ATCC VR-977 stock of Towne and of commonly used variants of AD169 in comparison with a novel, genetically more intact variant.

As shown in Fig. 1, the commonly used variants of Towne and AD169 arose from wild-type HCMV genomes (236 kb) via replacement of a region (U_L_/*b′*) at the right end of U_L_ (Cha *et al.*, 1996; Davison *et al.*, 2003b; Dolan *et al.*, 2004; Dunn *et al.*, 2003; Murphy *et al.*, 2003; Prichard *et al.*, 2001). These large-scale lesions were accompanied by additional mutations. AD169 has frameshifts in genes RL5A, RL13 and UL131A (Akter *et al.*, 2003; Davison *et al.*, 2003a, b; Yu *et al.*, 2002) and a substitution in gene UL36 that inactivates the encoded inhibitor of apoptosis (Skaletskaya *et al.*, 2001). Towne has frameshifts in genes RL13 and UL130 (Dolan *et al.*, 2004).

The Towne genome sequence has been determined (Dunn *et al.*, 2003; Murphy *et al.*, 2003) from a bacterial artificial chromosome (BAC) constructed from plaque-purified ATCC VR-977 (Marchini *et al.*, 2001). However, ATCC VR-977 is known to contain a mixture of two variants (Hahn *et al.*, 2003). One (Towne varRIT3 or Towne_short_; here called varS) is represented in the BAC and, as described above, lacks U_L_/*b′*. The other (Towne_long_; here called varL) is intact in U_L_/*b′*, and this region has been sequenced (Dolan *et al.*, 2004).

We determined the sequence of ATCC VR-977 by aligning data obtained from an Illumina Genome Analyzer (http://www.illumina.com) against a constructed reference sequence. To obtain the reference, the more reliable of the two varS BAC sequences (AC146851; Murphy *et al.*, 2003) was reorganized into a genome-equivalent arrangement after identifying the termini and removing insertions in gene UL32 and the origin of DNA replication. The varS reference was generated from this sequence by inserting the region containing genes IRS1–US12 from strain Merlin (AY446894; Dolan *et al.*, 2004) in place of the plasmid vector, which had replaced these genes during generation of the BAC. The varL reference was then constructed by inserting the U_L_/*b′* sequence (GenBank accession no. AY446869; Dolan *et al.*, 2004) into the varS reference in place of the duplication that had originally replaced U_L_/*b′*.

The Illumina data were derived from whole cell DNA extracted from human fetal fibroblasts infected with ATCC VR-977, and assembled and viewed using Maq and Maqview (Li *et al.*, 2008; http://maq.sourceforge.net). A total of 47.1 % of the 5 079 235 sequences (50 nt each) aligned with the derived varL consensus and the coverage was 516 reads per nt. Test assemblies using appropriate references confirmed that ATCC VR-977 contains both varL and varS, and showed that the whole population has frameshifts in genes RL13, UL1, UL130, US1 and US9. In addition, a 346 bp deletion in gene UL40 was present in almost the whole population, though detection of very sparse data from the deleted region indicated that a small proportion (much less than 1 %) of genomes might be intact. This deletion implies that varL and varS do not encode the gene UL40 signal peptide sequence, which contributes to natural killer (NK) cell evasion by upregulating human lymphocyte antigen-E (Tomasec *et al.*, 2000). Eighteen clear single nucleotide polymorphisms (SNPs) were identified, but these could not be assigned to particular variants. In addition to the presence of U_L_/*b′*, and excluding the duplicate copies of the inverted repeats, ATCC VR-977 differed from the varS BAC at 40 loci consisting of 34 substitutions and six insertions. By referring to sequences available for other HCMV strains, it was possible to infer which sequence was mutated at 25 loci. The BAC was assessed as being mutated at 23 and ATCC VR-977 at one, with the remaining substitution corresponding to an SNP.

In contrast with ATCC VR-977, in which varL has retained U_L_/*b′*, all commonly used AD169 stocks lack this region. Therefore, it was of interest that one of us possessed an AD169 stock (varUC) that reputedly contained U_L_/*b′* sequences. N. Lurain had received varUC at the University of Chicago from K. Thompson, who in turn had obtained it from M. Beem in 1981 at the same institution. No documentation was available on its history, but it was thought to have undergone at least 50 passages since its acquisition. Initial studies (N. Lurain, unpublished data) had demonstrated that varUC plaques were similar to those of strain Toledo (Kemble *et al.*, 1996), which contains U_L_/*b′* (Cha *et al.*, 1996), appearing as clusters of refractile, rounded cells, rather than the well-separated, elongated cells characteristic of commonly used AD169 variants. Also, sequencing had revealed the presence of U_L_/*b′* genes in varUC, specifically UL146 and a region containing the 3′ end of UL144 and the 5′ end of UL140 with a 3.2 kb deletion encompassing the intervening genes UL142 and UL141. Moreover, genotyping data from microarray experiments had indicated the presence of all U_L_/*b′* genes except UL142 and UL141 (J. García-Ramírez, D. Foster, L. Buehler, N. Lurain & P. Ghazal, unpublished data). These findings implied that varUC is either an AD169 variant that has retained most of U_L_/*b′* or another strain entirely.

In order to distinguish between these possibilities, several genes that are mutated in commonly used AD169 variants or that vary greatly between HCMV strains were amplified by PCR from DNA extracted from a stock of cell-released varUC and sequenced; U_L_/*b′* was also sequenced in its entirety. These data were compared with the published genome sequences of two AD169 variants. One of these was varUK, for which the sequence (X17403) was derived by Chee *et al.* (1990) and updated (BK000394) by the correction of errors and the insertion of a 929 bp region that is absent from certain stocks (Dargan *et al.*, 1997; Mocarski *et al.*, 1997). The other was varATCC, for which the sequence (AC146999) was derived by Murphy *et al.* (2003) from a BAC generated from plaque-purified ATCC VR-538 (Yu *et al.*, 2002). In the 16976 bp of the 28780 bp determined for varUC that were comparable with varUK and varATCC, all three genomes were closely similar. The presence of U_L_/*b′* in varUC, except for the previously characterized 3.2 kb deletion, was confirmed. This deletion is predicted to result in lack of expression of the UL144, UL142 and UL141 proteins, and expression of the UL140 protein with the C-terminal eight residues replaced by 71 residues specified by a reading frame that overlaps UL144.

The partial information obtained was consistent with varUC being an AD169 variant, and formed the basis for deriving the complete genome sequence from Illumina data derived from DNA extracted from pelleted cell-free virions. A reference was constructed from the varUK sequence, utilizing the partial varUC data to amend differences, and inserting U_L_/*b′* in place of the duplication that had originally replaced U_L_/*b′*. A total of 92.4 % of 6 264 332 sequences (50 nt each) aligned with the derived varUC consensus, and the coverage was 1267 reads per nt. Test assemblies and PCR experiments demonstrated the absence of the U_L_/*b′* deleted form characteristic of varUK and varATCC and the 929 bp deleted form present in some varUK stocks. The 3.2 kb deletion in U_L_/*b′* was confirmed as being a feature of the entire population, and a 3.7 kb deletion in *c′*/U_S_, which affects genes IRS1, US1 and US2, was detected in the majority of the population. Test assemblies also showed that the entire varUC genome population contained the mutations in RL5A, RL13, UL36 and UL131A present in varUK and varATCC. Four clear SNPs were identified.

Given the apparent existence of a vast number of differentiable HCMV strains (e.g. Bradley *et al.*, 2008; Rasmussen *et al.*, 2003), the high degree of sequence similarity between the three variants and the sharing of several mutations in common, confirmed that varUC is an AD169 variant. Differences due to insertions included the presence of U_L_/*b′* in varUC, length variations in the tandem repeat in *a*/*a′* and heterogeneity in several non-coding polynucleotide tracts. Substitutions were identified at 54 loci (Table 1), with over half (29) in *bac*/*b′a ′c′* and the adjacent sequence at the left end of U_L_, and 32 in protein-coding regions (five synonymous and 27 non-synonymous). By referring to sequences available for other HCMV strains, it was possible to infer which sequence was mutated at 42 loci. A total of 36 mutations were specific to a single variant: nine to varUC and 27 to varATCC. Each of the remaining six mutations was present in pairs of variants: one in varUC/varUK, two in varATCC/varUC and three in varATCC/varUK.

Given the lack of historical information, it is not possible to reconstruct fully the lineages that led to the three AD169 variants. AD169 was isolated at the National Institutes of Health (Bethesda, MD, USA) from the adenoids of a 7-year-old girl and passaged 14 times in human fibroblast cells to yield a stock named NIH 76559 (Rowe *et al.*, 1956). The lineage that led to varUK was initiated by researchers at St George's Hospital Medical School (London, UK), who obtained NIH 76559 in 1960 and passaged it 40 times in human fibroblast cells. The resulting virus was used to make batches of a potential vaccine by 16–24 additional passages (Elek & Stern, 1974). The varUK sequence was determined from a set of plasmid clones generated from a plaque-purified derivative of one of these passages (Oram *et al.*, 1982). The route by which varATCC was derived from NIH 76559 is less clear, but it seems likely that it originated from an exclusively US lineage, since a US researcher, W. A. Chappell, deposited the stock with the ATCC. The ATCC has declined to reveal to us when this occurred, but has distributed AD169 at least since 1973 (e.g. Smith & de Harven, 1973), and now markets it as ATCC VR-538. The relatively large number of mutations specific to varATCC is consistent with the impressive numbers of passages to which AD169 was subjected in early years in some US laboratories (e.g. 232 passages by Vonka & Benyesh-Melnick, 1966). If varATCC indeed originated from a purely US source, a schema of the type illustrated in Fig. 2 may be proposed. However, resolution of the details is confounded by the potential persistence of mutations (including those that may have arisen in the ancestor of any two or all three variants) to different extents in subsequent lineages, and to the fact that the varUK and varATCC sequences originated from molecular clones made from plaque-purified viruses and therefore do not necessarily represent whole populations.

Like many other passaged HCMV strains, the Towne and AD169 variants are mutated in gene RL13 and one of the three genes in the UL128 locus (UL128, UL130 and UL131A) (Akter *et al.*, 2003; Dolan *et al.*, 2004; Hahn *et al.*, 2004). This implies strong selection during passage in human fibroblasts against the encoded functions, which are involved in cell tropism (reviewed by Sinzger *et al.*, 2008a). Towne varS and the AD169 variants are also mutated in U_L_/*b′*, as is strain TB40/E, which is frameshifted in UL141, with a derivative additionally lacking UL145 and UL144 (Dolan *et al.*, 2004; Sinzger *et al.*, 2008b; Tomasec *et al.*, 2005). The patterns of mutation suggest that more than one gene in this region (UL145, UL144, UL142, UL141 or UL140) may be selected against, though not as strongly as RL13 and the UL128 locus. It is not immediately apparent why expression of these genes might be deleterious. The proteins encoded by UL142 and UL141 are involved in evasion of NK cell function, the former by downregulating MICA, which is a ligand for the activating receptor NKG2D (Chalupny *et al.*, 2006; Wills *et al.*, 2005), and the latter by sequestering CD155 (PVR), which is a ligand for the activating receptors CD226 (DNAM-1) and CD96 (TACTILE) (Tomasec *et al.*, 2005). The UL144 protein activates NF-*κ*B in a TRAF6-dependent manner, causing upregulation of the chemokine CCL22 (MDC) (Poole *et al.*, 2008), and also inhibits T cell proliferation by binding CD272 (BTLA) (Cheung *et al.*, 2005).

We have contributed towards the characterization of variants of HCMV strains Towne and AD169, so that biological data may be assessed with greater rigour. The sequence of Towne ATCC VR-977 confirmed the presence of two major variants (varL and varS) and extended knowledge of their shared mutations. A novel AD169 variant (varUC) was shown to be genetically more intact than varUK and varATCC and may be a new tool in the hands of HCMV researchers.

## Figures and Tables

**Fig. 1. f1:**
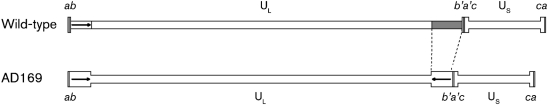
Schema for the derivation of the major lesion in the genome of commonly used AD169 variants from a wild-type virus genome; lengths are shown to scale. In the wild-type genome, the long and short unique sequences (U_L_ and U_S_; shown as thinner structures) are flanked by inverted repeats (*ba*/*b′a′* and *ca*/*c′a′*; shown as thicker structures). As indicated by the dashed lines, the AD169 genome was generated by replacing a region at the right end of U_L_ (U_L_/*b′*; shaded grey) by an inverted duplication of a region from the left end of the genome (arrow). This resulted in U_L_ becoming shorter by 15 kb (19 genes) and *b/b′* becoming longer by 10 kb (six genes and part of another). A similar phenomen occurred in the derivation of the Towne varS genome, with U_L_ becoming shorter by 13 kb (15 genes) and *b*/*b′* becoming longer by 11 kb (seven genes and part of another).

**Fig. 2. f2:**
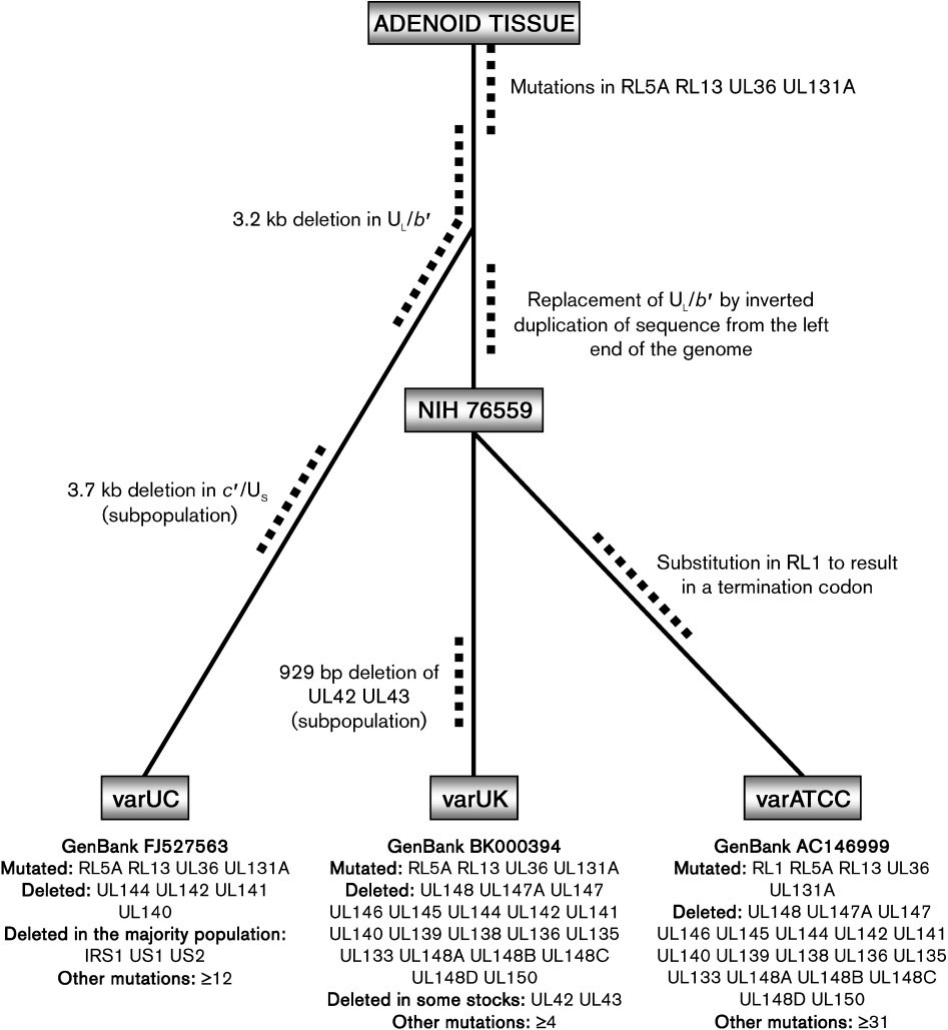
Schema for the derivation of AD169 variants from the original clinical material, based on the assumption that varATCC was generated from a US source and not from importation of varUK. Mutations in RL5A, RL13, UL36 and UL131A (and perhaps other, undetected mutations resulting in amino acid substitutions or affecting non-coding sequences) arose during the 14 passages that led to NIH 76559 and were inherited by all three lineages. Later on during the 14 passages, the deletion of U_L_/*b′* occurred, and this was inherited by varUK and varATCC from NIH 76559. The 3.2 kb deletion in U_L_/*b′* that characterizes varUC occurred either at an intermediate stage as a precursor of the larger deletion or in the separated lineage. Genes that are detectably disabled, and other mutations whose effects are unknown (Table 1), are listed below each variant.

**Table 1. t1:** Nucleotide substitutions in AD169 variants nd, Substitutions where the mutant and non-mutant residues could not be distinguished; cr, synonymous substitutions or substitutions not located in protein-coding regions; sub, non-coding and synonymous substitutions.

Nucleotide*	**Mutated variant**	**Region affected**	**Effect†**
971	varUC	cr	sub
1059	varATCC	RL1	L→S
1080	varATCC	RL1	T→R
1095	varATCC	RL1	Q→R
1166	varATCC	RL1	C→S
1270	nd	RL1	Q•H
1403	varATCC	RL1	C→R
1672	varATCC	RL1	I→M
1749	varATCC	RL1	S→ς‡
1925	varATCC	RL1	W→R
2337	varATCC	cr	sub
2781	varATCC	cr	sub
2898	varATCC	cr	sub
3337	varATCC	cr	sub
5080	varUC	RL5A§	sub
8318	varATCC/varUK	RL10	N→D
27980	varATCC	UL23	sub
29374	varATCC	UL24	F→L
42128	varATCC	UL32	E→G
56856	varUC	UL44	A→S
98223	varATCC	cr	sub
103266	varUC	UL70	H→Y
108681	varUC	UL74A	sub
114378	varATCC	UL78	S→F
118556	varUC	UL82	D→A
118730	varATCC/varUK	UL82	R→P
134412	varATCC/varUK	UL89	A→S
136946	varATCC	UL93	A→D
145916	varATCC	UL99	F→S
155061	varATCC	UL105	sub
160678	varATCC	UL111A	K→R
175085	varUC	cr	sub
191003	nd	*b′*	sub
191021	nd	*b′*	sub
191043	nd	*b′*	sub
191071	nd	*b′*	sub
191113	nd	*b′*	sub
191146	nd	*b′*	sub
191277	nd	*b′*	sub
191334	nd	*b′*	sub
191336	nd	*b′*	sub
191588	nd	*a′*	sub
191635	nd	*a′*	sub
191743	varATCC	*a′*	sub
192163	varATCC	*c′*	sub
192244	varUC/varUK	*c′* (TRS1/IRS1)	sub
192325	varATCC/varUC	*c′* (TRS1/IRS1)	sub
192434	varATCC	*c′* (TRS1/IRS1)	S→G
192723	varATCC	*c′* (TRS1/IRS1)	L→P
192770	varUC	*c′* (TRS1/IRS1)	V→L
192983	varUC	*c′* (TRS1/IRS1)	T→P
197200	varATCC	cr	sub
215658	varATCC/varUC	US23	C→Y
224225	varATCC	US29US30	L→P

*Location in the varUC genome sequence (FJ527563; 231781 bp). This excludes the duplicate copies of the inverted repeats (i.e. *ab* and *ca*) and 12 substitutions (nt 222147–223855) in a region of the varATCC sequence that was replaced during construction of the BAC. This replacement appears to have originated from a strain other than AD169 (probably Toledo). †For non-synonymous substitutions, an arrow indicates the change from the non-mutant to the mutant amino acid residue. Where it was not possible to distinguish the non-mutant and mutant residues, the alternatives are separated by a filled circle. ‡Termination codon indicated by ς. §Frameshifted in all variants and therefore considered non-coding.
